# Energy expenditure during physical work in cold environments: physiology and performance considerations for military service members

**DOI:** 10.1152/japplphysiol.00210.2024

**Published:** 2024-08-29

**Authors:** Erica A. Schafer, Christopher L. Chapman, John W. Castellani, David P. Looney

**Affiliations:** ^1^Thermal and Mountain Medicine Division, United States Army Research Institute of Environmental Medicine (USARIEM), Natick, Massachusetts, United States; ^2^Oak Ridge Institute for Science and Education, Oak Ridge, Tennessee, United States; ^3^Military Performance Division, United States Army Research Institute of Environmental Medicine (USARIEM), Natick, Massachusetts, United States

**Keywords:** cold stress, energetics, exercise, metabolism, oxygen uptake

## Abstract

Effective execution of military missions in cold environments requires highly trained, well-equipped, and operationally ready service members. Understanding the metabolic energetic demands of performing physical work in extreme cold conditions is critical for individual medical readiness of service members. In this narrative review, we describe *1*) the extreme energy costs of performing militarily relevant physical work in cold environments, *2*) key factors specific to cold environments that explain these additional energy costs, *3*) additional environmental factors that modulate the metabolic burden, *4*) medical readiness consequences associated with these circumstances, and *5*) potential countermeasures to be developed to aid future military personnel. Key characteristics of the cold operational environment that cause excessive energy expenditure in military personnel include thermoregulatory mechanisms, winter apparel, inspiration of cold air, inclement weather, and activities specific to cold weather. The combination of cold temperatures with other environmental stressors, including altitude, wind, and wet environments, exacerbates the overall metabolic strain on military service members. The high energy cost of working in these environments increases the risk of undesirable consequences, including negative energy balance, dehydration, and subsequent decrements in physical and cognitive performance. Such consequences may be mitigated by the application of enhanced clothing and equipment design, wearable technologies for biomechanical assistance and localized heating, thermogenic pharmaceuticals, and cold habituation and training guidance. Altogether, the reduction in energy expenditure of modern military personnel during physical work in cold environments would promote desirable operational outcomes and optimize the health and performance of service members.

## Graphical abstract

**Figure:**
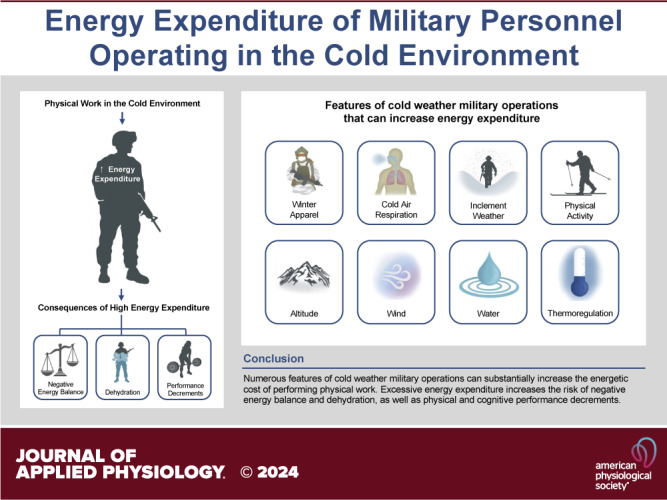

## INTRODUCTION

Cold weather military operations test the limits of human survival (1). Effective execution of military missions in such environments requires highly trained, well-equipped, and operationally ready service members (2). Therefore, it is imperative to understand the physiological impacts of cold-weather operations on modern military personnel. In particular, understanding the metabolic energetic demands of performing physical work in extreme cold conditions is critical for individual medical readiness.

**Figure 1.**
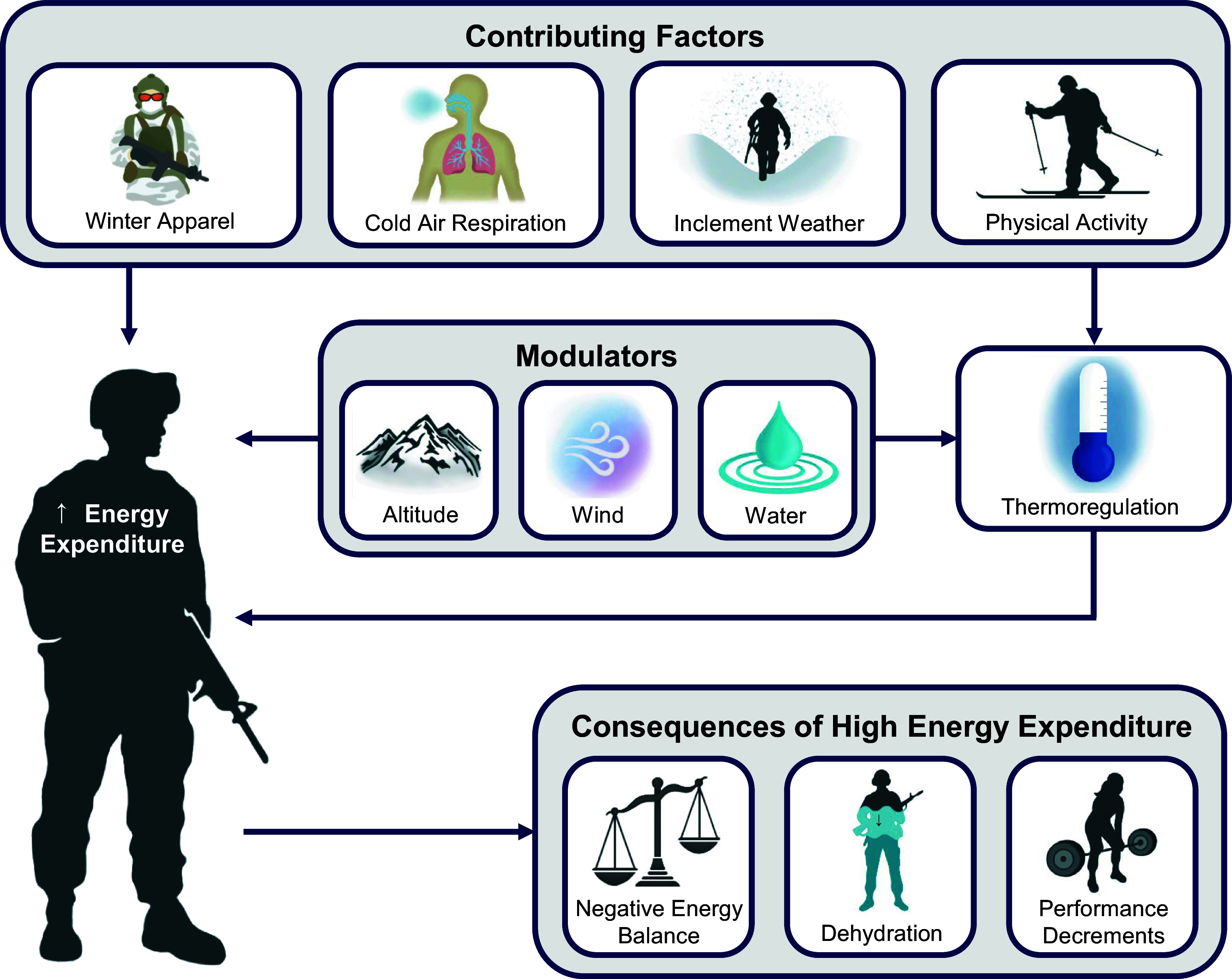
Factors influencing energy expenditure of physical activity during cold-weather military operations.

Metabolic energy expenditure requirements of military service members can be considerably high due to the heightened physical demands of everyday tasks (3). This is further complicated by the addition of thermal stress from the cold operational environment (4). Military operations conducted in cold environments are metabolically costly due to many factors (Fig. 1). Key characteristics of the cold operational environment that cause excessive energy expenditure in military personnel include thermoregulatory responses, winter apparel, inspiration of cold air, inclement weather (e.g., snow, ice), and activities specific to cold weather (e.g., snow shoveling, skiing) (3). The combination of cold with other environmental stressors that modulate cold stress, including altitude (5), wind (6), and wet environments (7), exacerbates the overall metabolic strain on military service members. Excessive energy expenditure is associated with numerous operational and physiological consequences (3), including physical (e.g., loss of motor control and dexterity) and cognitive performance decrements secondary to chronic negative energy balance (8) and cold-induced dehydration (9). Such consequences may compromise the readiness and lethality of individual military personnel and, ultimately, overall mission completion.

In this narrative review, we provide evidence of the extreme metabolic energy costs of performing militarily relevant physical work in cold environments. Additionally, we describe the key factors specific to cold environments that explain these additional energy costs along with modulating environmental factors that compound the associated metabolic burden. Finally, we discuss the physiological consequences of excessive metabolic energy expended during physical work in cold environments as well as potential countermeasures to be developed to aid future military personnel.

## ENERGY COST OF EXERCISING IN THE COLD

To gain a thorough understanding of the energetic responses associated with exercising in a cold environment, it is necessary to discuss the individual components of energy expenditure, as well as different measurement techniques and their respective advantages and limitations. Total daily energy expenditure (TDEE) is defined as the total amount of energy utilized to maintain the vital physiological functions of an individual throughout a 24-h period (10). TDEE is a function of metabolic processes that cause heat production or thermogenesis. Whole body thermogenesis is related to basal metabolism as well as activity, diet, and thermoregulation (11). Basal thermogenesis results from the physiological processes needed to sustain human life in stress-free, thermoneutral conditions (12). Activity thermogenesis results from performing exercises (running, resistance training, etc.) (13) as well as nonexercise activities such as daily living, fidgeting, and other spontaneous physical actions (14). Diet-induced thermogenesis, also known as the thermic effect of food (TEF), is composed of the energy expended to digest food (obligatory diet-induced thermogenesis) as well as the cost of sympathetic activation of brown adipose tissue, skeletal muscles, and potentially other organs secondary to food ingestion (facultative diet-induced thermogenesis) (15). Cold-induced thermogenesis is defined as an increase in energy expenditure above the basal metabolic rate (BMR) to balance the increased heat lost to the cold environment and is classified as either shivering or nonshivering (16). Cold-induced shivering thermogenesis involves the involuntary, asynchronous contraction of skeletal muscle to transiently produce heat in an effort to maintain body temperature with relatively little external work (17). In contrast, cold-induced nonshivering thermogenesis results from activating brown adipose tissue (11) as well as skeletal muscle via protein leak (18, 19), calcium cycling (20), and potentially other mechanisms. All components of TDEE are influenced by both internal and external factors, including age, sex, body size and composition, genetics, and previous history of exposure to the environment, with activity energy expenditure being further influenced by movement economy and exercise training (21).

**Figure 2.**
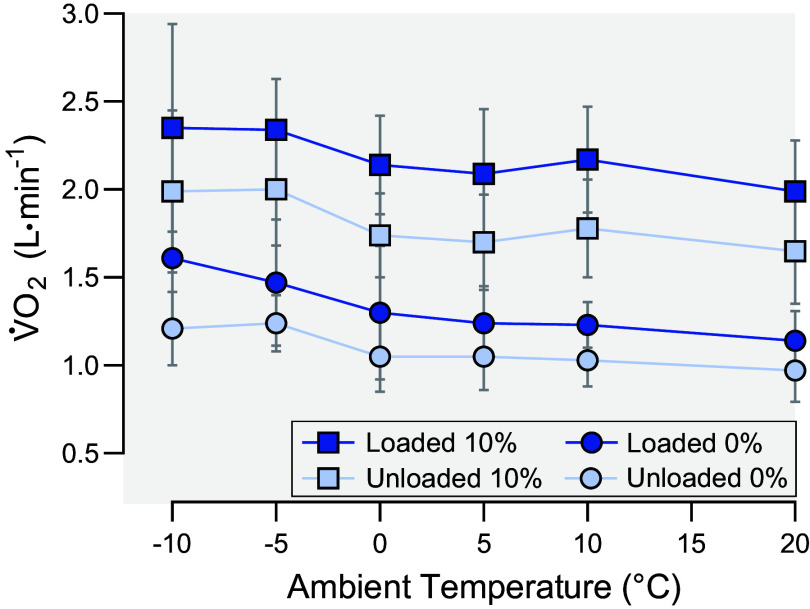
Effect of ambient temperature on oxygen uptake (V̇o_2_) during treadmill walking while unloaded or loaded (18.2-kg backpack) on 0 and 10% grades from Hinde et al. (27).

Metabolic energy expenditure is typically quantified using either indirect calorimetry (22) or the doubly labeled water (DLW) technique (23). The interested reader is referred to previous publications where these techniques have been comprehensively reviewed (23). Briefly, indirect calorimetry involves the collection and analysis of expired respiratory gases produced during metabolism (22). During metabolic steady state, the respiratory exchange ratio (RER) of carbon dioxide production (V̇co_2_) to oxygen uptake (V̇o_2_) measured in expired gases is assumed to reflect the ratio of CO_2_ produced to O_2_ consumed at the cellular level, known as the respiratory quotient (RQ), and consequently, the mixture of substrates oxidized (24). The percentages of energy provided by glucose and fatty acid oxidation and the energy equivalent of oxygen are derived from RQ using the computational approach outlined in Péronnet et al. (25). Indirect calorimetry via metabolic cart is most suitable for laboratory settings where the participant is confined within a small space (e.g., treadmill walking) and for relatively shorter measurement periods (26). Notably, Hinde et al. (27) used indirect calorimetry to demonstrate the effects of progressive cold stress on oxygen uptake (V̇o_2_) during loaded and unloaded treadmill walking on level and uphill grades (Fig. 2). The increased V̇o_2_ at lower temperatures emphasizes that more metabolic energy is required to perform the same militarily relevant exercise in the cold compared to a temperate condition, which if not accounted for, could lead to earlier exhaustion. Although portable indirect calorimetry systems are generally accurate (e.g., within ± 0.10 L V̇o_2_·min^−1^ of gold standard Douglas Bag method) (28) and reliable (e.g., coefficient of variation of 4.5%) (29) for indirect calorimetry outside of the laboratory, cold ambient temperatures are generally below normal operating ranges (e.g., <10°C). However, laboratory-based studies could utilize indirect calorimetry via a room calorimeter, in which specialized equipment within the room would collect expired respiratory gases without the need for accessory equipment that would be more sensitive to environmental changes (30).

Unlike indirect calorimetry, DLW (23) allows researchers to measure energy expenditure in unrestricted conditions (e.g., field studies) over longer periods of time (up to 21 days) (31, 32). The DLW technique assesses whole body water and CO_2_ turnover with deuterium (^2^H) and H_2_^18^O, respectively, with CO_2_ production calculated from the difference in the rates of isotope elimination (3). Energy expenditure is then calculated from CO_2_ production as RQ (3). The optimal testing period for healthy adults is between 4 and 21 days (33), with generally shorter testing periods for military nutrition studies (34). An advantage of DLW is that individuals are free to move around large spaces without restrictions imposed by the instruments (e.g., face masks), which contributes to the improved suitably of using DLW in the field (32). Additionally, the test-retest reliability of the DLW technique is also exceptional (e.g., bias ± SD, −5 ± 73 kcal·day^−1^) (35). However, the disadvantages associated with the DLW technique include that it is technically challenging, expensive (e.g., cost of isotopes and analysis equipment), and lacks the granular temporal resolution of indirect calorimetry (i.e., energy expenditure cannot be calculated on a minute-by-minute basis) (36). Consequently, DLW may not accurately assess brief bouts of high-intensity exercise or changes in substrate utilization (31).

Table 1 displays the energy expenditures of the United States and other international military personnel quantified by the DLW technique during cold-weather operations. The environments endured by military personnel in these studies ranged from a high of 21°C air temperature during warmer than expected winter operations in wet conditions (39) to extreme cold ambient temperatures of −39°C (45) or environments with harsh winds in excess of 90 kph (38). Notably, the data in Table 1 indicate that energy expenditure can exceed more than five times that of BMR during these cold operations, which is more than double the work rate that has been traditionally used to classify heavy work (47). In addition to the increased metabolic costs of thermoregulation, the elevated energy expenditures are caused by several factors specific to military operations, including prolonged intense work (e.g., ski marches and significant load carriage), restrictions that impair mobility during physical activity, uneven and arduous terrain, and inclement weather. These factors and their impact on energy expenditure are described in more detail in subsequent sections. For example, several studies report personnel carrying packs weighing ∼45 kg (∼55% of body mass) during 6–10 h of ski marching with work-to-rest ratios of 50:10 min that resulted in average energy expenditures between 4,732 and 6,155 kcal·day^−1^ (8, 46, 48). These elevated energy expenditures typically result in severe energy deficits between an average of −3,782 and −1,422 kcal·day^−1^, resulting in significant reductions in body mass that have been reported to be on average between −5.6% and −1% (37–43, 48, 49). An additional observation is the relative lack of data on female service members, with only three studies including females in their study population (37, 39, 46). More research is needed (see future directions) to understand the effects of cold operations on energy expenditure in female military personnel given that the U.S. opened all combat roles to female service members in December 2015 (50).

**Table 1.** Energy expenditure of cold weather military field training and exercises measured by the doubly labeled water technique

Reference	*n*	Country and Service	Location	Duration, day	T_min_, °C	T_max_, °C	Description of Work	EE, Kcal/day
Ahmed et al. (37)	6 M/4 F	Canadian Armed Forces Class A Reservists	Canadian Force Base Meaford	4	−22	−2	Basic military qualification course (land) under winter weather conditions	5,099 (746)/4,642 (589)
Burstein et al. (38)	18 M	Israeli Infantry Soldiers	Northern Israel	12	3	12	Prolonged periods of movement on foot over mountainous terrain with load carriage	4,281 (721)
Castellani et al. (39)	29 M/20 F	U.S. Marine recruits	Parris Island Marine Corps Recruit Depot, SC	2.25	3.6/6.3	18.8/21.4	54-h rigorous field training exercise termed “The Crucible”	6,142 (191)/4,732 (143)
DeLany et al. (40)	16 M	Special operations Soldiers	Camp Ethan Allen Training Center, Jerico, VT	25	−1.1	16.1	Traveled 46–48 km over 25 days with load carriage up to 45 kg and conducted local reconnaissance missions with no load	3,400 (260)
Hoyt et al. (41)	23 M	U.S. Marines	Marine Corps Mountain Warfare Training Center, Bridgeport, CA	11	−15	13	Winter military training course at elevation between 2,220 and 2,550 m, including skiing, snowshoeing, and 10-km biathlon	4,919 (911)
Hoyt et al. (42)	6 M	Special forces Soldiers	Mt. Rainier, Mt. Rainier National Park, WA	6	−14	−7	5 consecutive days of activity followed by 1 rest day; elevation between 2,550 and 3,100 m and load carriage of 31.1 (3.6) kg	4,558 (566)
Hoyt et al. (43)	14 M	U.S. Marines	Quantico, VA	10	−5	10	U.S. Marine Corps Infantry Officer Course Field Exercise in cold/wet conditions	5,378 (678)
Jones et al. (44)	10 M	First Royal Canadian Regiment	Iqaluit, Baffin Island, Canada	10	−35	−25	Work included skiing, snowmobiling, ice fishing, hunting, and igloo building	4,317 (927)
King et al. (45)	10 M	U.S. Army Soldiers	Fort Greely, AK,	10	−39	−8	Artic Warrior Field Training Exercise	4,253 (477)
Margolis et al. (8)	21 M	Norwegian Soldiers	Sør-Varanger, Kirkenes, Norway	7	−26	6	54-km ski march, 6–10 h/day, 50:10 work-to-rest ratio, traveled about 20 km per day with ∼45-kg load carriage	6,140 (394)
Margolis et al. (46)	42^a^	Norwegian Soldiers	Skjold, Norway	4	NR	NR	Arctic Military Training, 51-km ski march	6,155 (515)

Values as means (SD). Data for a given study are disaggregated by sex where possible. EE, energy expenditure; F, females; M, males; NR, data not reported; T_max_, maximum ambient temperature; T_min_, minimum ambient temperature. ^a^Male and female ratio not specified for this subset (*n* = 71 males and *n* = 2 females in total sample).

## CONTRIBUTING FACTORS

The cold operational environment is modified by a myriad of environmental stressors. Key factors that explain the excessive metabolic energy demands of exercising in cold environments include thermoregulation of cold stress, winter apparel, cold air respiration, inclement weather, and cold weather activities. In this section, we will discuss these factors and the underlying physiology driving increased energy expenditure in the cold operational environment.

### Thermoregulation of Cold Stress

Human metabolism increases under cold stress due to the need for thermoregulation. Thus, a brief discussion of the acute physiological responses to cold stress is warranted. Humans defend and regulate core temperature within a narrow range irrespective of the external environment with resting values between 36 and 38°C (51, 52). Human thermoregulation is controlled by a combination of afferent and efferent processes to maintain thermal balance (4). These processes were demonstrated by the seminal studies of Hammel (53) and Cabanac and Massonnet (54), which displayed the superimposed thresholds of heat loss and heat production in both humans and small mammals. The most significant afferent inputs of the thermoregulatory system, core and skin temperature, contribute to metabolic heat production by a ratio of ∼3.6:1, respectively (55). Cold stress comprises a multifaceted integration of factors related to environmental conditions (i.e., absolute temperature, rate of temperature change, duration of exposure, and exposure medium such as dry air, moist air, water immersion, and contact with ice) and human morphology (i.e., body surface area and location of exposure) (56). A superfamily of transient receptor potential (TRP) channels known as thermoTRP channels that are sensitive to polymodal stimulation serve as molecular temperature sensors in the human skin, with channels such as TRPM8 involved in innocuous cold-sensing located at a higher density in the periphery compared to the body core (57–59). This distribution is considered physiologically advantageous for defending core temperature in cold environments and allows for a rapid, reflex cutaneous vasoconstriction to occur as the primary autonomic mechanism (60). Recent studies provide evidence that normal cold sensing is encoded by both absolute temperature and changes in temperature (61). Accordingly, when skin temperature is reduced, thermoTRP channel activation results in the conduction of afferent signals along neural pathways to the preoptic anterior hypothalamus where they are integrated to produce neural signals along efferent neural pathways to cutaneous blood vessels, brown and beige adipose tissue, and skeletal muscle, which has been described in detail (62). This results in reduced convective heat loss via increased tissue insulation of the body shell due to reduced blood perfusion of the skin (60). There is an orderly effect to the recruitment of autonomic and behavioral thermoeffectors whereby thermoeffectors are thought to be engaged according to their relative physiological (i.e., metabolic) cost (63). As such, during cold stress, reflex cutaneous vasoconstriction occurs before changes in thermal behavioral and metabolic rate (64), whereas thermal behavior occurs subsequent to reflex cutaneous vasoconstriction but before increases in metabolic rate (65). The result of a combination of lowered skin temperature and reflex cutaneous vasoconstriction is the decreased temperature of peripheral nerves and muscle tissues, which can lead to significant impairment and reduced efficiency (66). For every 10°C decrease in local temperature, peripheral nerve conduction velocity decreases by ∼15 m·s^−1^ (67), while maximal muscle power and mechanical efficiency fall by 3% for every 1°C decrease in muscle temperature (68). This results in notable performance decrements such as impaired ability to complete fine-motor tasks (69) and altered motor unit recruitment patterns resulting in weaker muscle contraction (70).

Cold-induced thermogenesis involves an increase in metabolic rate during cold stress occurring without physical activity (e.g., walking) and comprises nonshivering and/or shivering thermogenesis. Collectively, these mechanisms typically allow for up to a three- to fivefold increase in energy expenditure above BMR (16). Nonshivering thermogenesis is primarily stimulated by mild cold and may elicit up to a 30% increase in energy expenditure (71, 72). Early reports have concluded that nonshivering thermogenesis is predominantly the result of a sympathetically mediated increase in oxidative metabolism of brown and beige adipocytes with elevated mitochondrial uncoupling protein-1 allowing for heat generation (73, 74). However, more recent literature has indicated that the pectorals and deep central neck muscles may be major contributors to heat production via nonshivering thermogenesis, while brown and beige adipocytes make up only a small percentage (75). Shivering thermogenesis is the greatest contributor to heat generation in cold humans and involves intense rhythmic, involuntary contractions of the skeletal muscle whereby little external work is performed (72). Thus most of the energy expended is released in the form of heat (72). While shivering may begin at the onset of cold exposure, the magnitude of shivering depends on the severity of cold stress (i.e., changes in core and skin temperature). In cold air, the metabolic rate can reach 200–250 W (76). However, in cold water, metabolic heat production increases to ∼350 W due to lower core and skin temperatures increasing the thermoregulatory drive. Maximal shivering for most individuals is ∼40% of maximal oxygen uptake (V̇o_2max_) or five times BMR (77, 78). The maximal shivering rate reported in the literature is 763 W, which occurred during 12°C water immersion when the volunteer’s core temperature approached 35°C (79). Shivering may also occur during exercise, primarily during low-intensity activities, but is associated with discomfort, fatigue, and reduced locomotion (76, 80).

Involuntary muscle contractions are fueled by carbohydrates, lipids, and proteins, which are delivered to muscle tissue via blood circulation (81). Fuel selection is determined by shivering pattern (e.g., shivering frequency and intensity) (82). Substantial variation has been observed between studies aiming to analyze fuel selection during shivering (83). Haman et al. (84) noted a fourfold increase in fat oxidation compared to that of carbohydrates and found fat to account for a greater percentage of total heat production. In a separate study, Haman et al. (85) also reported a significant increase in the utilization rate of carbohydrates during moderate-intensity shivering as compared to low intensity, while the rates of lipids and proteins remained constant. The relative contribution of each substrate to total heat production also changes with shivering intensity, with percent carbohydrates displaying a 1.4-fold increase, while percent lipids and proteins decrease significantly (85).

There are several notable modifiers of the thermoregulatory effector responses to cold, which have been comprehensively reviewed elsewhere (4). First, differences in anthropometrics and body composition are attributed to explaining most of the variability in thermoregulatory responses to cold between individuals. Certain anthropometric measurements (e.g., body surface area, body surface area to volume ratio) are critical to predicting thermoregulatory responses of individuals of varying sizes and can be measured directly using three-dimensional body surface scans or estimated using population-specific height- and weight-based formulas (86, 87). In general, greater body surface area-to-mass ratios cause greater declines in body temperature during cold exposure (88). However, the thermal resistance to heat conduction (i.e., insulation) differs between body tissues, with fat providing the highest thermal resistance and unperfused skeletal muscle also contributing significantly to total body insulation (4, 89). Second, although not fully understood, differences in morphology largely, but not fully, explain sex differences in the thermoeffector and perceptual responses to cold (90, 91). For example, females tend to have greater body surface area-to-mass ratios compared to males (87), which would increase convective heat loss, and less skeletal muscle mass, which decreases the insulative capacity to mitigate heat loss (92). However, when anthropometric measures are controlled for, there appears to be similar increases in relative cold-induced metabolic heat production between sexes (78, 93–96). Third, exertional fatigue can cause decrements in thermoregulatory effector responses during and following physical work, which is referred to in the literature as “thermoregulatory fatigue” and may lead to hypothermia (97). Hypothermia remains a risk during military operations and training, including a recent report finding that there were an average of 75.8 diagnosed cases of hypothermia per year (379 total) among the active component of the U.S. Armed Forces between 2018 and 2023 (98). Fourth, older adults (>60 yr) may be less tolerant to cold environments compared to their younger counterparts due to decreases in peripheral vasoconstriction, physical fitness, heat conservation, and reduced temperature threshold for the onset of shivering (4).

### Winter Apparel

**Figure 3.**
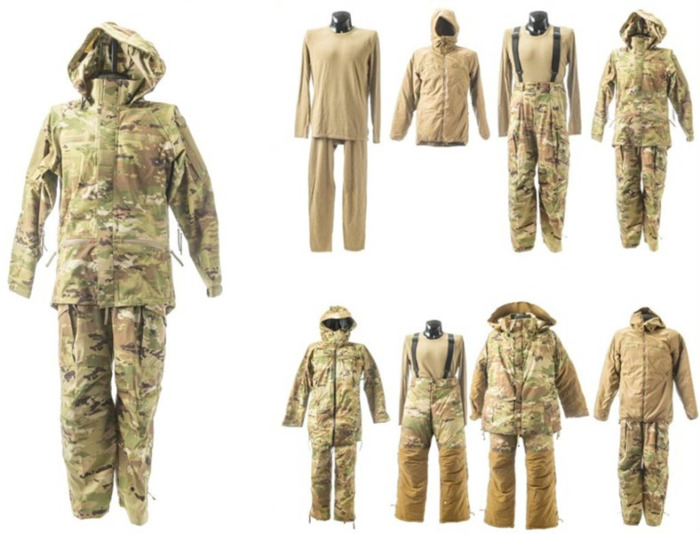
U.S. Army’s Cold Temperature and Arctic Protection System (105).

Proper cold weather clothing reduces thermoregulatory demands while protecting against freezing and nonfreezing injuries but imposes its own metabolic costs on an exercising individual. Cold weather clothing, which is designed to mitigate heat loss and wetness through increased insulation and impermeable membranes (99), is heavier and more restrictive than the typical garments donned in temperate and hot environments. Thus, these features of cold-weather clothing create additional mass that must be moved during exercise and also have the potential to greatly limit an individual’s mobility (100). Generally speaking, the extent to which additional mass affects the metabolic costs of activity increases the further away from the body’s center of mass it is carried (i.e., more distal anatomical locations have higher metabolic costs) (101). While the hands and feet are the least economical anatomical locations to carry added mass (101), these locations also have the greatest need for cold protective clothing due to their relatively higher susceptibility to frostbite (102). Modern tactical boots, typically required for military field exercises, result in 7%–10% higher walking energy expenditure versus running shoes (103). Bulky cold weather clothing can limit joint range of motion, resulting in a “hobbling effect” (2, 100). For example, wearing additional cold weather clothing layers of the U.S. Army’s Extended Cold Weather Clothing System (ECWCS) restricts bending at the waist and shoulder mobility as well as causes greater trunk flexion and reduced arm swing during walking (104). Fortunately, the U.S. Army recently developed the Cold Temperature and Arctic Protection System (CTAPS) to improve Soldier lethality and readiness during cold weather operations (Fig. 3) (105), although these advantages still need to be quantified by thermophysiological research. While foot travel is still possible in these scenarios, impaired or eliminated movement patterns, such as restricted arm swing, reduce the metabolic economy of movement (106). Another consideration is that multilayer clothing systems create increased frictional resistance from layers sliding over one another during movement, ultimately requiring individuals to expend more metabolic energy to overcome this limitation (100). This bulkier clothing also poses aerodynamic disadvantages (107) because the metabolic cost of overcoming air resistance during locomotion directly increases with a greater surface area of the clothed individual (108). However, these effects may only be problematic during high-speed movements (skiing, snowmobiling, etc.) (109) or extreme wind conditions. Finally, “overdressed” service members insulated by excessive cold protective clothing risk additional metabolic strain due to thermoregulatory demands, initially from evaporative cooling mechanisms (i.e., sweating) (100), and subsequently from cold-induced thermogenesis after clothing becomes wet with sweat. The additional mass, restricted mobility, increased frictional resistance, greater surface area, and potential overdressing effects contributed by cold weather clothing combined are an extra metabolic burden that must be considered with the thermoregulatory benefits provided by the added insulation. Selecting the appropriate cold-weather clothing ensemble for a given scenario will limit unnecessary metabolic strain from excessive layering as well as shivering and nonshivering thermogenesis.

### Cold Air Respiration

Breathing cold dry air can stress the respiratory system and result in higher metabolic demand during exercise even in thermoneutral ambient temperatures (110). Inspired cold air must be rewarmed to body temperature and ∼100% relative humidity in the lower airways (111). This rewarming causes heat and water loss from the airways, resulting in respiratory epithelium dehydration (112). Prolonged cold air respiration may lead to airway constriction, shortness of breath, and hyperventilation, thereby increasing demands placed on respiratory muscles to contract faster and more forcefully (27). As such, Sandsund et al. (110) observed a modest increase in V̇o_2_ between −15°C and 32°C conditions (29.8 ± 3.4 vs. 28.7 ± 2.9 mL·kg·min^−1^, respectively) during 30 min of steady-state treadmill exercise at 55%–60% V̇o_2max_. In addition to concerns with cold air-induced bronchoconstriction, King et al. (45) observed a high prevalence of upper respiratory symptoms during a cold weather field training exercise in Alaska where, of the 96 U.S. Army Soldiers, ∼50% experienced nasal or sinus congestion, ∼60% with rhinorrhea, and ∼30% with coughing. High ventilation-cold weather exercises, such as ice skating and Nordic skiing, can also result in exercise-induced bronchospasm, which presents symptoms similar to an acute asthma attack including chest tightness, coughing, shortness of breath, and wheezing (112). This is important to consider as the presence of respiratory symptoms could have a negative impact on physical performance, therefore jeopardizing mission success (113). Unfortunately, effective countermeasures for mitigating the deleterious effects of cold air, such as reducing exercise intensity and covering one’s mouth (114), are not always feasible for military personnel.

### Inclement Weather

**Figure 4.**
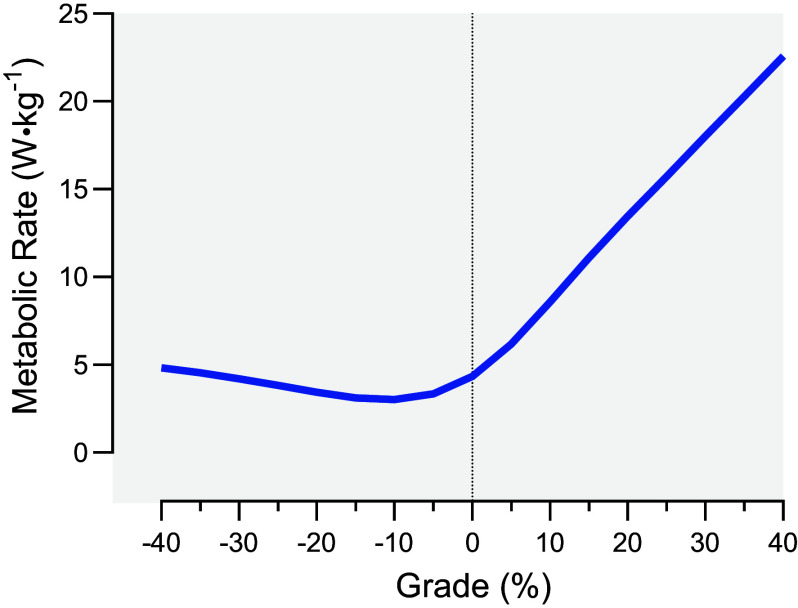
Estimated metabolic rate when walking in military boots at 1.34 m·s^−1^ across extreme surface grades (119, 194).

Inclement weather such as snowfall greatly impedes exercise performance (7, 115–118). Traveling by foot over deep snow is particularly difficult as metabolic costs increase as a function of sinkage into the snow (∼2.84 times normal walking metabolic rate with 10 cm of sinkage) (115). Walking over slippery surfaces expends ∼70% more metabolic energy than paved roads or treadmill-like surfaces (116). In addition, icy and slippery surfaces increase the risk of slips, falls, and other operational accidents (117). Prolonged hiking (1–5 h) in rain, wind, and cold without adequate rainwear can increase metabolic rate by 40% due to shivering thermogenesis while reducing dexterity and strength capabilities (7). Extreme gradients, commonly found in Arctic mountain regions, incur considerable metabolic costs on both uphill and downhill slopes (Fig. 4; Ref. 119). While one can maintain a sustainable metabolic rate in these winter weather conditions by moving slower, the total metabolic energy expended over a given route will increase if pacing falls below the most economical speeds (120), and physical tasks often must be completed within specific time limits (121). Moreover, the extended time to completion means prolonged cold exposure for the exercising individual, thereby increasing the risk of cold injuries.

### Cold Weather Activities

**Figure 5.**
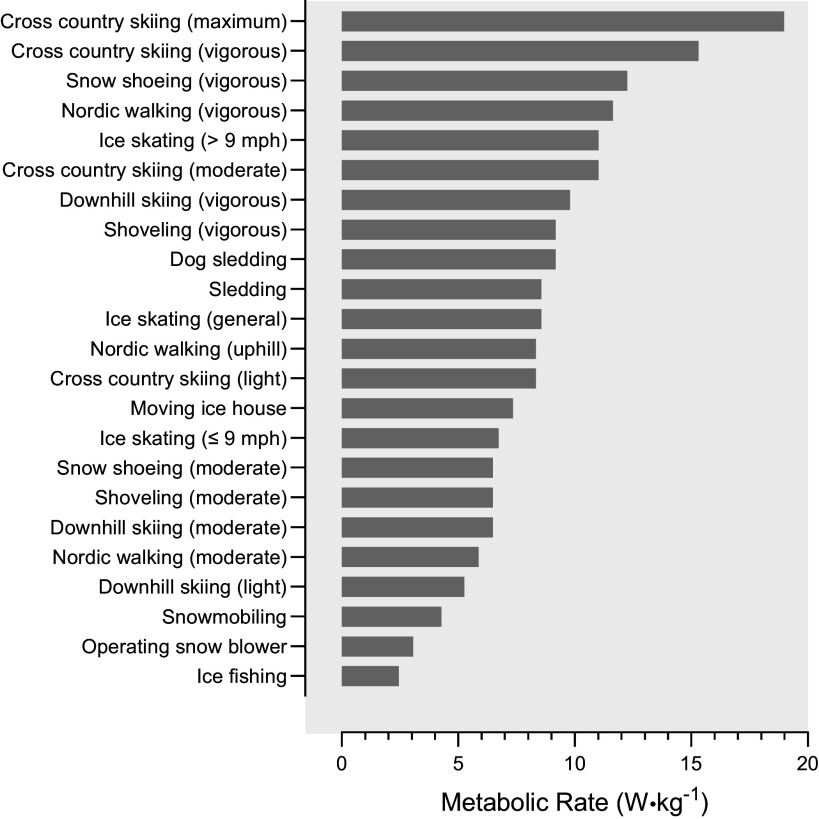
Metabolic rates of cold weather activities converted from the American College of Sports Medicine Compendium of Physical Activities (122).

While inclement cold weather impairs exercises typically performed in thermoneutral conditions, many physical activities unique to cold environments have substantial metabolic energy requirements (Fig. 5; Ref. 122). Due to greater activation of upper and total body musculature, activities such as cross-country skiing, shoveling, and snowshoeing elicit higher energy costs than many modes of human locomotion typical of thermoneutral environments. These activities also require the carriage and use of accessory equipment such as shovels, skis, ski poles, and snowshoes. Shoveling snow requires coordinated activation of lower body, back, and upper body musculature and is associated with musculoskeletal injury risk to the lower back and shoulder (123). Depending on the size of the shovel blade and position of the handle, shoveling can have a significant impact on energy expenditure and performance (123), with a large square blade and secondary handle being optimal for energy expenditure (124). Cross-country skiing at vigorous intensities can elicit the highest energy expenditure (∼20 W·kg^−1^) of cold-weather activities (122). Electromyographical data indicate additional activation of upper extremity musculature during skiing, specifically the triceps brachii, pectoralis major, teres major, and latissimus dorsi muscles, to repeatedly plant the ski pole and propel the body forward (125). As a result of the added muscle activation, aerobic energy cost can be 5%–9% higher when skiing with poles as opposed to without (126). Snowshoeing, a practice intended to ease physiological and biomechanical strain during locomotion over snowy terrain, can actually impose a significant metabolic cost, given that snowshoeing can impose gait changes and limit movement efficiency, thus driving increased walking economy (127). Footwear used during cold-weather activities (i.e., skis, skates, snowshoes) also contributes to increases in energy expenditure as they add additional weight to be moved by the lower extremities. Modest increases in V̇o_2_ have been reported regarding the addition of winter footwear, wherein a 100-g increase in footwear weight increases V̇o_2_ by ∼0.7%–1.0% for a given work rate (128). Unfortunately, the removal of winter footwear and accessory equipment to mitigate increases in energy expenditure would compromise efficient mission completion and exacerbate the risk of cold injury.

## ENVIRONMENTAL MODULATORS

There are several environmental factors that modulate the magnitude of cold stress incurred by military service members during cold weather operations. Additionally, these factors have both independent and interdependent factors that may heighten energy expenditure during work in cold environments. These environmental modulators are discussed below.

### Altitude

Military personnel deployed to cold environments are likely to encounter changes in altitude, which can greatly increase physiological and metabolic demands independent of the cold temperature. Generally, high altitude is considered to be terrain elevation greater than 3,050 m above sea level (5) where ambient temperatures decrease by ∼6.5°C for every 1,000 m of elevation above sea level (129). Average TDEE in military personnel has been measured to be in excess of 4,558 kcal·day^−1^ when conducting cold operations at altitudes between 2,220 and 3,100 m (Table 1; Refs. 41, 42). During acute exposure to altitude, hypobaric hypoxia lowers the partial pressure of oxygen in the blood and tissues, thereby resulting in compensatory increases in ventilation, sympathetic activation, and cardiac output to meet the increased metabolic demands of the body at rest and during physical activity (130).

**Figure 6.**
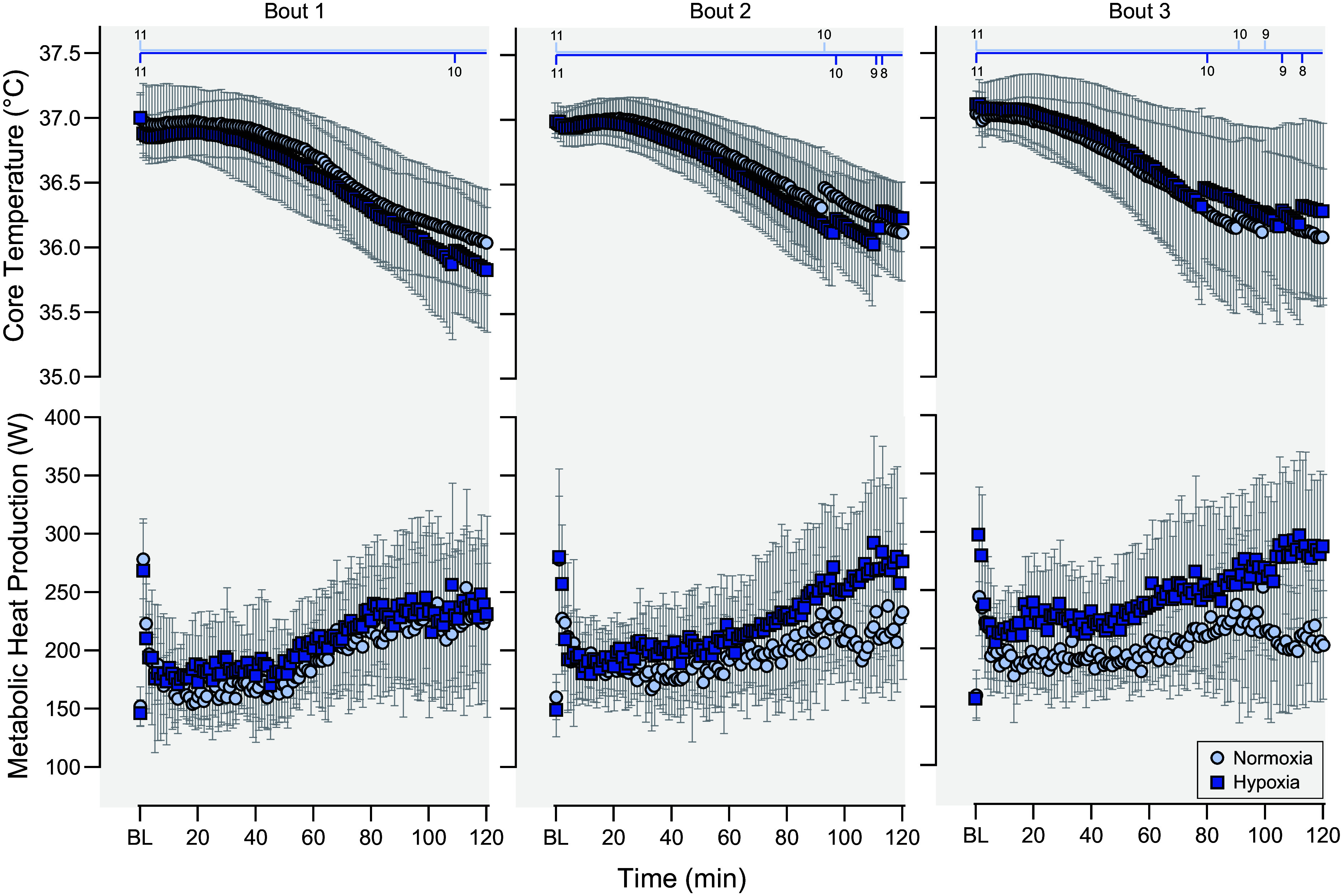
Effect of normobaric hypoxia compared to normobaric normoxia on core temperature (rectal) and metabolic heat production during 3 successive 120-min bouts of cold-water immersion (20°C) that were each separated by 120 min for rewarming. A given bout was terminated early when core temperature reached 35°C and is represented by the number of participants remaining at the *top* (light blue, normoxia; dark blue, hypoxia). Values as means with 95% confidence intervals; *n* = 11 men. Data from Keramidas et al. (146), with permission.

Reductions in body mass are a common observation in high altitude research and may be reflective of loss of body fat and lean mass associated with hypocaloric diet, diet manipulation, or decreases in total body water that occurs through altitude-induced diuresis, respiratory water loss, and potentially sweating (131–133). However, reductions in total body water have not been consistently observed at altitude (134, 135). Additionally, body mass losses at altitude are contributed to by higher BMR, increased energy demands of physical activity, appetite suppression, and possible intestinal dysfunction (136). Studies in lowlanders venturing to high altitudes have shown increased BMR that peak over the first 2–3 days after arrival by ∼27% (137, 138) with sustained increases occurring for at least 3 wk (138). While physical activity and/or hypocaloric diets led to greater reductions in body mass at altitude, a meta-analysis by Dünnwald et al. (139) found that 0.4- to 2.5-kg reductions in body mass occurring at altitude persisted even when caloric intake was matched or increased relative to output. Moreover, hypoxia does not appear to alter substrate utilization to the total energy yield during exercise at the same relative intensity compared to when performed in normoxic conditions (140). Studies have also demonstrated that V̇o_2max_ decreases by 10% for every 1,000-m increase in elevation above 2,000 m (5, 141–145). Notably, hypoxia progressively augments energy expenditure during prolonged cold stress, as demonstrated by Keramidas et al. (146) using a well-controlled experimental model of normobaric hypoxia during three bouts of 120-min cold water immersion (Fig. 6). In addition to performance decrements, acute altitude exposure is associated with cognitive decrements (e.g., psychomotor performance, reaction time, and logical reasoning) (147) and acute mountain sickness, the latter of which may begin at 700 m above sea level (130). These interactions are especially relevant to military operations given that it is not always possible to prepare for such outcomes during rapid deployment to mountainous regions (5).

### Wind

**Figure 7.**
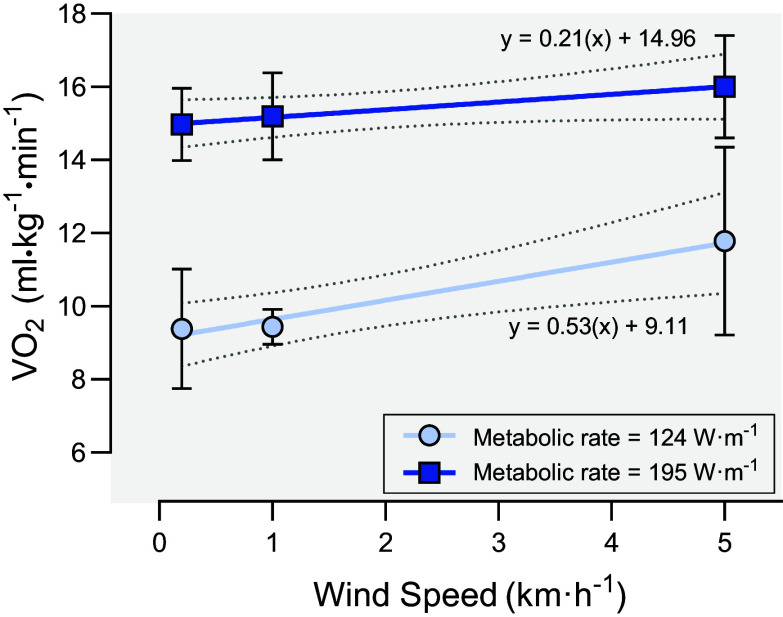
Effect of wind speed on oxygen uptake (V̇o_2_; mean ± SD) during incline treadmill walking performed at lower (metabolic rate = 124 W·m^−1^) and higher (metabolic rate = 195 W·m^−1^) relative intensities in −10°C environmental temperature. Line of best fit from weighted linear regression presented with 95% confidence bands. Data from Mäkinen et al. (150), with permission.

Wind is a common characteristic of cold weather operations that can greatly intensify the cold stress experienced by military personnel. In studies of energy expenditure during cold military operations, wind has been documented to be sustained in excess of 90 kph (38) and be so harsh that mission completion is prohibited (Table 1; Ref. 42). Greater wind speeds enhance convective heat loss and reduce the insulative value of protective cold-weather clothing (76, 148), thereby causing marked reductions in local skin temperatures, especially the face and distal regions (i.e., hands and feet), and causing earlier and more intense shivering to occur (149). Mäkinen et al. (150) reported ∼25% higher V̇O_2_ values during treadmill walking with wind speeds of 5.0 m·s^−1^ compared to no wind (0.2 m·s^−1^) during exercise intensity set to a metabolic rate of 124 W·m^−1^, but the effect of wind was attenuated at higher work rates (195 W/m^2^) as noted by a ∼7% increase in V̇o_2_ at the higher wind speed (Fig. 7) (150). Energy expenditure may also be increased by the additional effort required to overcome the opposing force of wind resistance (6, 151). The classic study by Pugh (6) quantified the effect of horizontal wind force on the energetic cost of both walking and running. When calculated as ΔV̇o_2_, the energy cost of overcoming wind resistance while running at middle distance speeds (6.0 m·s^−1^) was 7.5% and 13.6% for sprinting (10.0 m·s^−1^) (6). This may, theoretically, be exaggerated by bulky cold-weather clothing as it may increase the force of aerodynamic drag (152).

### Wet Environments

The cold operational environment can be further complicated by water in the form of rain, snowfall, immersion (i.e., head out of water), or submersion (i.e., head under water). Water significantly increases cold stress during immersion, compared to air, due to the heat transfer coefficient in water being ∼25-fold greater and, through conduction and convection, leads to a two to four times greater heat loss during immersion compared to air of the same temperature (153–157). There is a profound acute response to cold water immersion due to the cold shock response, including marked increases in heart rate, total peripheral resistance, blood pressure, and hyperventilation, as well as reductions in cerebral blood flow velocity (158–160). Submersion of the face evokes the diving reflex causing parasympathetic activation and bradycardia through stimulation of the trigeminal nerve, which can cause autonomic conflict with the sympathetic stimulation from the cold shock response and increase the risk of arrhythmia (76).

The physical properties of water (i.e., the density, compressibility, pressure, and specific heat capacity) also present challenges to the physiological adjustments to movement during water immersion (161). Thus external and internal work is increased when moving in water, thereby increasing energy expenditure, as individuals must overcome the hydrodynamic resistance that comprises the sum of three simultaneously produced drag components (162). These drag components are *1*) form (pressure) drag, which increases with the speed of movement, is most predominant at low to moderate speeds (<1.2 m·s^−1^), and depends on the wetted area of the immersed body; *2*) friction drag, which is determined by the effect of viscosity between water and the body and is dependent on the surface characteristics of the body; and *3*) wave drag, which increases as the speed of movement increases, is most predominate at higher speeds (1.8–2.0 m·s^−1^), and is characterized by the displacement and rising water against gravity that forms waves (162).

**Figure 8.**
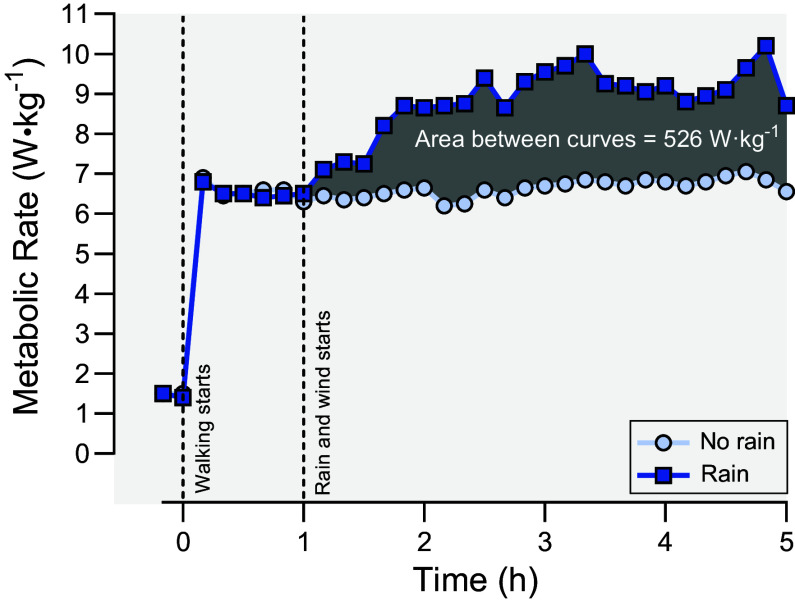
Effect of rain (∼7.4 cm·h^−1^) on metabolic rate while walking in a cold (5°C) and windy (8 km·h^−1^) environment. Five male participants walked for 5 h at a consistent speed (5.1 km·h^−1^) guided by pacing lights on a simulated hiking trail aligned with fans and overhead sprinklers. The first hour was without wind and rain while the remaining 4 h were with wind and either with or without rain; *n* = 5 healthy young males. Data from Thompson and Hayward (7), with permission.

Water also complicates the cold environment from clothes becoming wet. Heat loss in wet clothes has been reported to double compared to dry conditions at an air temperature of 5°C (163). During rainy conditions, energy demand may also increase via shivering or nonshivering thermogenesis. Ito et al. (164) reported significantly higher V̇o_2_, plasma lactate, and norepinephrine while running at an ambient temperature of 5°C in the presence of 40 mm·h^−1^ rainfall. The insulative capacity of clothing is reduced by water and the absorbed water within the cold-weather clothing poses additional mass to be carried by the individual (165, 166). Specifically, clothing becomes 1 g heavier for every 1 mL absorbed (167). The total difference in mass varies depending on the absorption capacity of the specific clothing materials. Bynum et al. (168) evaluated the metabolic rates of 10 men wearing 3 different clothing materials with varying insulative capabilities (vinyl, 0.43 clo; polyurethane, 0.61 clo; and neoprene, 0.76 clo) while immersed for 60 min in 20 and 28°C water. The metabolic rate was significantly higher for all three clothing materials during immersion in 20°C water, indicating less effective insulation (168). Additionally, Thompson and Hayward (7) reported a 40% increase in metabolic heat production as a result of significant shivering when walking during cold, wet, and windy conditions, which was also associated with decrements in strength and dexterity (Fig. 8).

Wet clothing can also occur when it becomes saturated with sweat as excessive sweating occurs due to a mismatch between work intensity and clothing insulation that causes heat gain despite cold ambient conditions. When clothing is saturated with sweat, just as with rain, it becomes ineffective at buffering the cold temperatures and may even freeze, both of which increase the risk of cold injury and hypothermia (9). Therefore, even in otherwise dry cold environmental conditions, the potential for the creation of a wet environment from excessive sweating is an important consideration during cold military operations (see *Winter Apparel*).

## CONSEQUENCES OF HIGH ENERGY EXPENDITURE

The result of failing to prepare for the high energy cost of operating in a cold environment is multifaceted. The primary outcome is a negative energy balance caused by a mismatch between high energy expenditure and low energy intake. During military operations, under-eating is prevalent even when sufficient calories are provided (169). This is a common occurrence in the cold operational environment for a number of reasons, including poor appetite, cold rations, short breaks to eat, eating by oneself, and poor palatability of most rations (170). Additionally, the amount of food available is limited to what can be carried, with any extra rations adding weight to the vest or rucksack (83). During work in cold environments, chronic negative energy balance imposes significant health risks to military personnel (171, 172), including systemic inflammation and catabolism of muscle protein, which promotes the loss of both fat-free and muscle mass, and associated performance decrements (172–174). It is well established that a 5%–10% loss of body mass is necessary to observe measurable performance decrements, specifically lower body power, with severe losses (e.g., 7%–10%) resulting in more dramatic declines (8, 175). However, Margolis et al. (8) identified progressive declines in lower body power in Norwegian Soldiers completing a 7-day winter training program after ∼2% reductions in body mass. Other factors of the study (i.e., strenuous physical activity, heavy load carriage) yielded more muscle damage, muscle soreness, and perceived fatigue, all of which impact performance (8).

**Figure 9.**
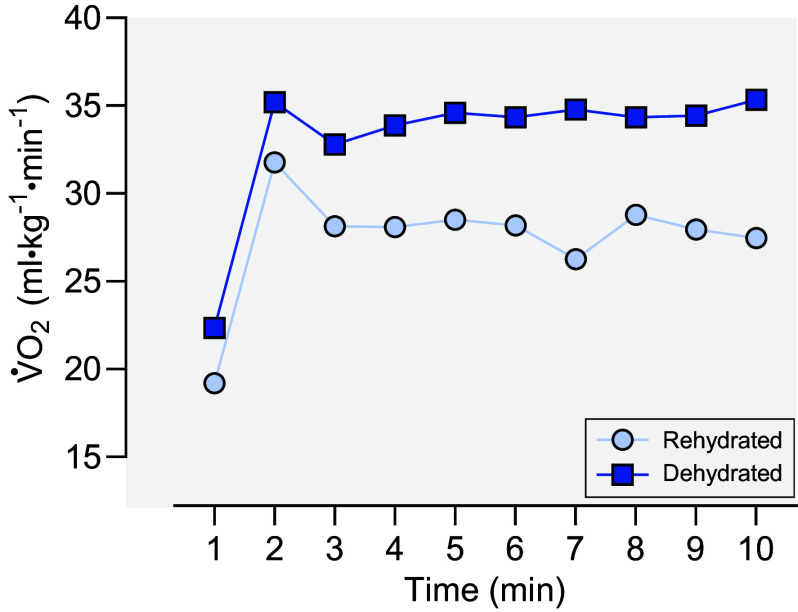
Effect of dehydration (3% body mass) on oxygen uptake (V̇o_2_) during a 10-min bout of submaximal exercise at 125 W at an environmental temperature of −15°C compared to when partially rehydrated (repletion of 1.8% of body mass loss) (177).

Another undesirable result of working in the cold operational environment is dehydration. Physical work in cold environments may cause fluid deficits of 3%–8% of body mass due to a number of factors, including a combination of cold-induced diuresis, blunted thirst, deliberate underdrinking, poor availability of water, respiratory water losses, and even high sweat losses when work intensity and/or insulation from excessive clothing increases the rate of heat storage (176). Furthermore, logistical or strategic concerns during missions may cause following rehydration schedules to be prohibitive during cold operations. Notably, dehydration of 3% body mass loss has been shown to elicit greater energy expenditure during submaximal intensity exercise in cold environments (Fig. 9) (177). However, the effects of dehydration in cold environments on physical performance can be somewhat contradicted in the literature (178) and is likely a function of several factors, such as the duration of exposure and the experimental model employed. For example, a dehydration of 3% body mass loss could reduce operational readiness in cold environments, as time to exhaustion in one study was reduced by 13% from an average of 12.2 min to 10.6 min (177). However, in the same study, no differences in V̇o_2max_ were observed between dehydrated and control conditions, indicating that aerobic capacity was not altered by dehydration in cold environments. It is worth noting that V̇o_2max_ may have remained stable due to the centralization of blood volume secondary to peripheral vasoconstriction. In studies of acute exposure, dehydration has been shown to have little to no effect on cognitive performance (e.g., working memory, attention, accuracy) in temperate or cold environments (179), despite the negative impact dehydration has in these parameters in hot environments (180). However, future research is needed to better understand the cumulative effects of dehydration over days on these parameters during prolonged cold weather operations.

## FUTURE DIRECTIONS

**Figure 10.**
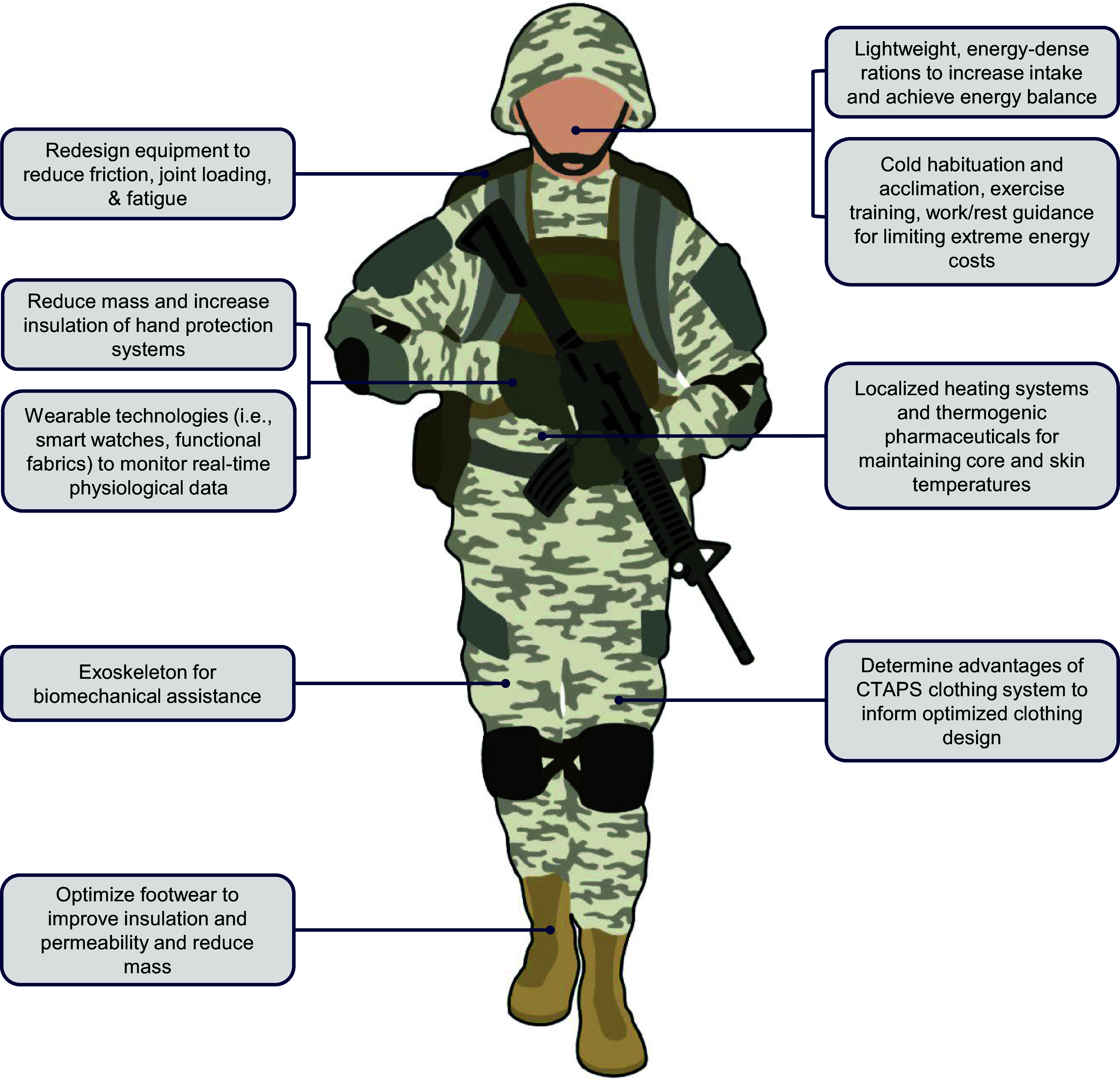
Potential countermeasures for future operational implementation to mitigate excessive energy expenditure and subsequent physiological consequences during cold-weather military operations. CTAPS, Cold Temperature and Arctic Protection System.

While the preceding sections outline why physical work in the cold is so energetically demanding, service members have numerous potential countermeasures being developed and refined for future operational implementation to ultimately prevent the aforementioned consequences (Fig. 10). The extreme metabolic costs of physical work in cold environments can be reduced through ergonomic enhancements to contemporary cold weather clothing and individual equipment. For instance, improvements to the length and angle of a snow shovel shaft can likely reduce lower-back joint loading (181). An extensive, recent review by Almqvist et al. (182) identifies future strategies for reducing ski-snow friction related to critical ski characteristics such as material, camber shape, stiffness, and base topography as well as their interactions with environmental features. Military personnel can also leverage the biomechanical advantages of load-carrying equipment such as the U.S. Army’s recently developed Modular Lightweight Load-carrying Equipment (MOLLE) 4000 rucksack, which is designed to distribute carried loads onto the hips away from the lower back and shoulders to reduce fatigue and enhance performance (183). Whether military members are provided metabolic and physiological advantages from wearing the new CTAPS cold weather clothing instead of the existing ECWCS remains to be determined experimentally. Identifying a hand protection system that optimizes both thermal protection and dexterity, two inversely related qualities, has been stated to be a critical objective by military personnel (2, 184). Similarly, footwear designers face difficult tradeoff analyses between user requirements that may be prioritized differently depending on the type of cold environment (e.g., insulation, mass, waterproof, vapor permeability) (185).

Advanced technologies designed to increase the metabolic efficiency of dismounted operations exist but must be adapted and validated for use in cold-weather military operations. For example, wearable physiological monitors that operate in extreme temperatures would be valuable for real-time work/rest guidance and early detection of cold weather injuries (2). Exoskeletons, body-worn mechanical devices that augment the user’s physical capabilities (186), have been developed for improving metabolic economy during walking and military load carriage (187) but must be shown capable of traversing snow, ice, and other winter weather conditions. Recent work demonstrated decreased metabolic costs of a repetitive lifting task in a 10°C environment when using a passive exoskeleton (188). Interestingly, the exoskeleton increased mean skin temperature and thermal sensation, which was attributed to the additional insulation provided by the device. Functional fabric clothing embedded with digital thermometer sensors provides a means of measuring local temperatures (189) and could be used for monitoring warning signs of frostbite. Localized heating devices positioned in key anatomical locations have been shown to increase skin temperatures during cold exposure and lead to improved manual dexterity and strength (190). However, active heating may not be sufficient to preserve manual dexterity during certain scenarios, such as cold immersion (191).

Unfortunately, addressing the negative energy balance resulting from prolonged cold weather military operations is not as simple as providing more rations to service members. Recent field studies (8, 46) reported that Soldiers participating in multiday Arctic military training only consumed approximately two-thirds of their combat rations. Margolis and Pasiakos (83) reviewed many of the present barriers to adequate energy intake in cold-weather military operations and highlighted potential solutions for future research and development. For instance, service members would benefit from expedited and efficient methods for thawing and rehydrating frozen rations during field operations, a common issue hindering energy balance achievement (83, 192). Given the higher energy yield from dietary fat (9 kcal/g) compared to carbohydrate and protein sources (∼4 kcal/g each), palatable high-fat military rations would deliver sufficient energy intake to service members experiencing appetite suppression during prolonged cold weather exercise (83). Although such rations would contain higher fat content (∼40% of total calories) than traditional rations (∼30% of total calories), they would still contain plentiful carbohydrates. Most military personnel perform prolonged lower intensity work during field operations rather than high-intensity exercises that require greater reliance on carbohydrate sources (193). Increased fat intake would ideally help minimize the energy deficit commonly experienced by military personnel during cold-weather field operations, provide exogenous energy to support performance, and spare use of glycogen. As metabolic energy expenditure increases with the amount of mass carried (194, 195), reducing the weight of military rations would decrease the energy costs as well as the consequent energy intake requirements of dismounted movements (83). Consequently, evaluating how a lighter weight energy-dense ration may impact energy intake and physical performance during cold weather military operations is of great importance. Overall, there are numerous ways that military rations can be optimized to better facilitate energy balance during cold-weather military operations that need to be explored.

Pharmacological strategies with thermogenic potential, such as the combination of ephedrine and caffeine (196), may be advantageous for maintaining body temperatures in cold weather with reduced reliance on shivering thermogenesis (2). Given shivering is associated with impairments to motor skills and coordination (197), these thermogenic aids could theoretically improve exercise economy at a given level of metabolic heat production. However, the ergogenic benefits along with the safety of such pharmaceuticals for use during strenuous physical work in the cold must be verified before widespread field implementation.

Cold weather habituation, exercise training, and work/rest guidance are possible strategies for limiting the extreme energy costs of dismounted military operations. Effective cold habituation practices decrease thermal sensation, cutaneous vasoconstriction, catecholamine release, shivering thermogenesis, and ultimately overall metabolic heat production (198). Cold acclimation has been shown to reduce shivering intensity without affecting total heat production, suggesting a compensatory increase in nonshivering thermogenesis (197). Gordon et al. (197) found that cold acclimation induced by 7 days of 1 h immersions in 14°C water improved cold tolerance and reduced shivering intensity by 36% without altering whole body heat production. These findings suggest that uncompensable cold acclimation/acclimatization strategies where core temperature is reduced repeatedly may be beneficial for cold military operational performance and survivability in these environments. However, to our knowledge, the efficacy of these strategies in mitigating increased energy expenditure or consequent operational performance decrements has not yet been investigated during military field operations. The benefits of cold weather exercise training can be seen in experienced skiers, who are more metabolically efficient due to their ability to simplify key movement patterns such as double poling by reducing unnecessary motions (199). The development of work/rest guidance for Arctic operations is inherently challenging since, in addition to changes in activity, clothing and environmental factors will differ from work periods when service members rest in insulated shelters for warmth. Service members must be able to work at a sufficient pace to complete their mission and stay warm outdoors without excessive sweating. While work/rest guidance for service members is currently much less defined for cold weather (200) than hot weather operations (201), an energetics-based decision aid that considers clothing, activity, and environmental factors for both work and rest periods would be of immediate value to military communities.

Finally, as demonstrated in Table 1, the effect of physical work in cold environments on energy expenditure has been predominately studied in men. Given the opening of combat roles to female military personnel in December 2015, further investigation into the effects of these environments on women is warranted (50) (Table 2). For example, the absolute load of gear and equipment (i.e., load carriage weight) cannot always be modified for women. Given that women are typically smaller than men, the energy expenditure of operational tasks may impose a greater metabolic burden on women compared to men, especially during periods of prolonged cold stress (202). Women may also require adjusted nutritional targets (i.e., total caloric intake, micronutrient intake) while operating in the cold environment to offset the risk of negative energy balance (50). Furthermore, not all sex differences in the thermoregulatory response to cold stress can be explained by differences in body morphology, and thus future investigation is required to better maximize health and performance in female military personnel working in cold environments (see *Thermoregulation of Cold Stress*) (203–205).

**Table 2.** Identified knowledge gaps regarding the energy expenditure of female military personnel operating in a cold environment

Identified Knowledge Gaps
Cold-weather clothing systems could be bulkier when worn by women. How would this effect the energy cost of overcoming wind resistance?
The absolute work rate and loads of militarily-relevant physical tasks typically cannot be adjusted for women. How do these interactions modulate energy expenditure in cold environments in women?
Given that women are typically shorter than men, does traversing snow of the same depth incur a higher energy cost?
Ergonomically incorrect equipment can increase energy expenditure throughout a cold-weather operation. How does the fitting of equipment impact the energy expenditure of female military personnel?
What feeding strategies can be developed for women that would account for adjusted caloric and nutritional targets to provide sufficient fuel for cold weather operations?

## CONCLUSIONS

Numerous features of cold weather military operations can substantially increase the energetic cost of performing physical work. The combined metabolic burden of increased thermoregulatory activity, added clothing, inspiration of cold air, traveling through inclement weather, and engaging in physically demanding cold weather activities, in addition to the possibility of altitude, wind, and wet environments, causes increases in energy expenditure that may compromise mission effectiveness. Excessive energy expenditure elevates the risk of physiological and operational consequences, in that negative energy balance and acute hypohydration result in physical and cognitive performance decrements. Such decrements may be mitigated by the application of enhanced clothing and equipment design, wearable technologies for biomechanical assistance and localized heating, thermogenic pharmaceuticals, and cold habituation and training guidance. Altogether, the reduction in energy expenditure of modern military personnel during physical work in cold environments would promote desirable operational outcomes and optimize the health and performance of service members.

## GRANTS

This research was supported in part by appointments to the Department of Defense (DOD) Research Participation Program administered by the Oak Ridge Institute for Science and Education (ORISE) through an interagency agreement between the U.S. Department of Energy (DOE) and the DOD. ORISE is managed by ORAU under DOE contract number DE-SC0014664.

## DISCLAIMERS

Approved for public release; distribution is unlimited. The opinions or assertions contained herein are the private views of the author(s) and are not to be construed as official or reflecting the views of the Army or the Department of Defense. Any citations of commercial organizations and trade names in this report do not constitute an official Department of the Army endorsement of approval of the products or services of these organizations. The authors have no conflicts of interest to declare. All opinions expressed in this paper are the authors’ and do not necessarily reflect the policies and views of DOD, DOE, or ORAU/ORISE.

## DISCLOSURES

No conflicts of interest, financial or otherwise, are declared by the authors.

## AUTHOR CONTRIBUTIONS

E.A.S., C.L.C., J.W.C., and D.P.L. conceived and designed research; E.A.S., C.L.C., J.W.C., and D.P.L. interpreted results of experiments; E.A.S., C.L.C., J.W.C., and D.P.L. prepared figures; E.A.S., C.L.C., J.W.C., and D.P.L. drafted manuscript; E.A.S., C.L.C., J.W.C., and D.P.L. edited and revised manuscript; E.A.S., C.L.C., J.W.C., and D.P.L. approved final version of manuscript.
